# Male sexual signaling and expected effects of hatchery-induced sperm competition vary with water depth at which whitefish are caught

**DOI:** 10.1093/cz/zoab007

**Published:** 2021-01-30

**Authors:** Giulia Perroud, David Nusbaumer, Christian de Guttry, Claus Wedekind

**Affiliations:** Department of Ecology and Evolution, University of Lausanne, 1015 Lausanne, Switzerland

**Keywords:** sexual signaling, sperm competition, whitefish

Salmonids like whitefish (*Coregonus* spp.) are often propagated in supportive breeding. Spawners are caught from their spawning locations, their gametes mixed, and the resulting offspring reared in a protected environment before being released into the wild. This procedure can affect sexual selection, for example, by enhancing the importance of sperm competition or by reducing the relevance of sexual signals. While it is often unclear how sperm competitiveness is affected by a male’s overall genetic quality, there is accumulating evidence that sexual signals reveal good genes and that mate choice based on such signals can increase offspring viability (Auld et al. 2019). Therefore, supportive breeding may affect the genetic variance and the mean genetic quality of next generations. We sampled whitefish from various locations along a depth gradient to test how male characteristics that are likely to affect sexual selection under natural conditions correlate with characteristics that affect hatchery-induced sperm competition. Whitefish are external fertilizers, and multi-male spawning and hence sperm competition is common under natural conditions. Mate choice is not sufficiently understood but could be based on breeding tubercles. These are small conical structures that grow on scales before the breeding season and fall off shortly afterwards. The size of breeding tubercles varies much among males and has repeatedly been found to correlate positively with offspring viability (Wedekind et al. 2001; Kekäläinen et al. 2010). Male–male dominance is typically dependent on body size (Auld et al. 2019) and could also be relevant in whitefish. Body size itself can reflect individual inbreeding coefficients (Su et al. 1996) and be an indicator of heritable genetic quality in small or structured populations (Neff and Pitcher 2008). In another fish with a somewhat comparable mating system, the size of breeding tubercles and male size was not correlated but could both be used to predict male reproductive success under close to natural conditions (Jacob et al. 2009). We study whitefish from Lake Hallwil (Switzerland). This lake has suffered so much from anthropogenic eutrophication that it is being artificially aerated since 1985. Three hatcheries around the lake are likely to have played a key role in maintaining the whitefish population, as concluded also from a recent mark–recapture experiment (Vonlanthen 2015). However, eutrophication combined with possible hybridization in hatcheries can have led to a speciation reversal (Vonlanthen et al. 2012) and may thereby have destroyed any genetic structure linked to water depth. Hatchery protocols now focus on maintaining overall genetic variance by pooling milt of many males before adding the mix to eggs of multiple females. Milt volume varies among sires, for example, because males often loose milt when being pulled up from deep locations (Figure 1), an effect that likely depends on how much the swim bladder is inflated by the change in pressure. This variance in milt volume is likely to affect the genetic variance that, in combination with the average genetic quality, may then affect the long-term survival of a population. The extent to which hatchery protocols affect genetic quality can be estimated by the correlations between male quality indicators and traits that affect hatchery-induced sperm competition, that is, sperm number, velocity, and longevity (summarized here as “milt potency,” see also Supplementary Material). Many breeding protocols are likely to promote genetic quality if male attractiveness or dominance are positively correlated to milt potency. If there are no such correlations or negative ones because of life-history trade-offs, hatchery-induced sperm competition is likely to reduce the average genetic quality in future generations. We sampled fish from various depths and determined their age, size, breeding ornamentation, and milt potency (see methods in the Supplementary Material) to test whether and how different male characteristics affect reproductive success in supportive breeding in a heavily managed population.

**Figure 1.**
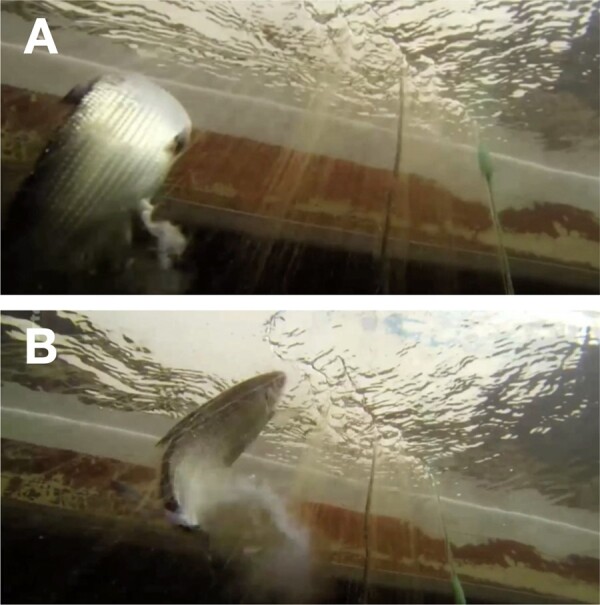
Male being pulled up from deep water and loosing milt just before being lifted into the boat. Photograph **A** also reveals the breeding tubercles on the male’s skin. Photograph **B** was taken about half a second after photograph A (©arte tv; reproduced with permission).

The samples taken at 25, 12, and 4 m did not differ in age (Fisher’s exact test, *N*_1_ = 54, *N*_2_ = 30, *N*_3_ = 14, *P = *0.58) nor in sex ratios (*P *=* *0.28). The males caught at different depths did also not differ in size (Supplementary Figure S1A)*.* However, the mean size of their breeding tubercles varied with depth (Supplementary Table S1 and Supplementary Figure S1B). The tubercles were significantly larger in males caught at 25 m depth than at 12 m depth (*post hoc* Tukey’s HSD test, *P = *0.03), while no such differences were observed in the other possible comparisons (*P* always *>* 0.12). Overall, larger males had larger breeding tubercles (Supplementary Table S1), but this relationship varied with location and was strongest in males caught at the deepest location (Supplementary Table S1 and Supplementary Figure S1C). Sperm velocity did not vary with water depth (measured at 12 versus 4 m for sperm characteristics), increased with body length (but not with age), and was not significantly revealed by the size of the breeding tubercles (Figure 2A–C and Supplementary Tables S2A and S3A). Mean sperm concentration did also not seem to vary with water depth, body length, or breeding tubercles (Figure 2D–G and Supplementary Tables S2 and S3B). Milt potency was significantly reduced in males caught at larger depth (Figure 2G and Supplementary Table S3B) and was overall not significantly linked to body length and breeding tubercles. However, there was a significant interaction between the effects of depth and breeding tubercles on milt potency (Figure 2H–J and Supplementary Table S2B).

**Figure 2.**
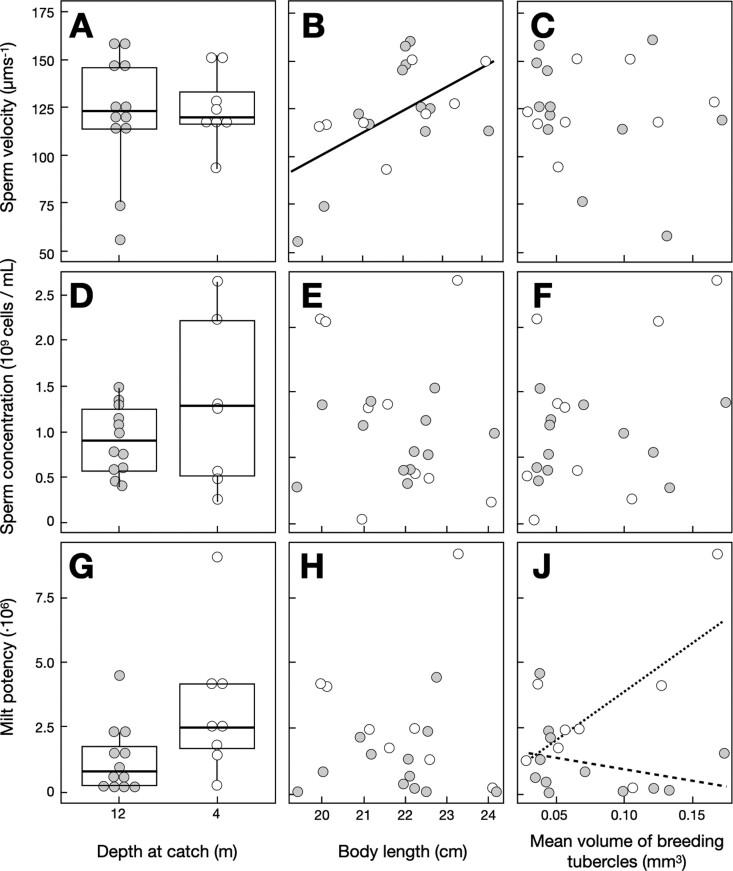
Sperm velocity (**A**–**C**), sperm concentration (**D**–**F**), and milt potency (**G**–**J**) versus water depth, body length, and mean breeding tubercles volume for fish caught at 12 m (gray symbols, dashed regression line) and fish caught at 4 m (open symbols, dotted line). Panels **A**, **D**, and **G** show Tukey boxplots with quartiles and whiskers. The regression line in panel **B** shows the significant relationship between body length and sperm velocity at both depths. The regression lines in panel **J** illustrate the significant interaction between depth and mean breeding tubercles volume on milt potency. No significant links could be observed in panels **C**, **E**, **F**, and **H**. See text for statistics.

The protocols of supportive breeding at Lake Hallwil and most (if not all) other Swiss lakes promote sperm competition (Wedekind et al. 2007). We studied what kind of males would be expected to profit from such hatchery-induced sperm competition. Our samples had similar size and age distributions. Nevertheless, fish caught at the deepest location had the largest breeding tubercles. It remains to be shown whether this difference in breeding ornamentation is due to genetics, environmental factors, or a combination of both. Intra-lacustrine diversification may have largely vanished during the eutrophication crisis (Vonlanthen et al. 2012) but could now be rebuilding itself so that phenotypic differences reflect genetic differences. Alternatively, if breeding tubercles are generally valid indicators of good genetic quality, fish caught in deeper locations could be on average of better genetic quality than fish caught in more shallow regions. However, the link between body length and breeding ornaments changed also with water depth, suggesting that either the information content of breeding tubercles does not depend on genetic quality alone, or that variance in genetic quality has location-specific effects on growth. We found that larger males had faster sperm, and that sperm velocity could not be predicted by breeding tubercles or water depth. There was no link between male size and milt potency, but we found an interaction between breeding tubercles and depth on milt potency. These observations suggest that there were no trade-offs between male dominance or mate attractiveness and investment into milt. On the contrary, males that are predicted to be successful under natural conditions, because they are large and/or well ornamented, seemed still able to invest more into high-quality sperm than small or less ornamented males, that is, there seemed to be positive correlations between the various fitness-relevant traits (Reznick et al. 2000). It remains to be tested whether such correlations are to be expected in non-managed populations or can be a consequence of hatchery-induced selection in previous generations. Our measure of milt potency was based on sperm number, velocity, and lifespan, but fertilization success can also be affected by the composition of the fertilization media (pH, ovarian fluids, etc.) and possibly further factors that may influence sperm motility. Based on our measurements, we conclude that the current protocol used in supportive breeding (mixing the milt of many males, then adding the mix to the eggs) may give large males and males caught from more shallow regions a reproductive advantage. Alternative breeding protocols would then affect hatchery-induced sperm competition differently: (i) When equalizing milt volume, hatchery-induced sperm competition would be driven by sperm velocity and sperm concentration. (ii) When equalizing cell counts, that is, taking sperm concentration into account, male reproductive success would be largely determined by sperm velocity. (iii) When equalizing milt potency, no type of male would be favored. However, minimizing the effects of milt potency, for example, in full-factorial crossings, would only minimize the loss of genetic variation over time. Genetic quality would not be promoted by such a breeding design. If breeding tubercles and body size are indeed indicators of heritable genetic quality, genetic quality could be promoted in hatcheries by giving large males and/or males with large breeding tubercles a reproductive advantage over small and/or poorly ornamented males. However, with declining effective population sizes (*N*_e_), minimizing the loss of genetic variance become increasingly important. Small *N*_e_ therefore require an optimization between minimizing the loss of genetic variance and minimizing the loss of genetic quality in order to maximize a population’s long-term survival probability. In conclusion, the consequences of hatchery-induced sperm competition are different for whitefish males caught at different water depths and at different body sizes. If variance in milt potency is ignored in the breeding protocols (as currently in the study population), males caught from more shallow locations are favored over males caught from deeper locations. Breeding protocols that would be based on equalized milt volume or even equalized sperm counts per male would give males with high sperm velocity a reproductive advantage. In our study population, these would be the large males. Any hatchery-induced variance in male reproductive success could be avoided, for example, in full-factorial breeding designs that minimize mean kinship within the next generation. However, minimizing the loss of genetic variance can reduce average genetic quality, because elevating the reproductive success of large and/or well ornamented males can positively affect the mean viability of the next generation.

## Funding

This work was supported by the Swiss National Science Foundation [31003A_182265].

## Supplementary Material

Supplementary material can be found at https://academic.oup.com/cz.

## Conflict of Interest

The authors declare no conflicts of interest.

## Supplementary Material

zoab007_Supplementary_Data

## Notes

Accepted Letter to Editor
