# Supplementary material for: Male sexual signaling and expected effects of hatchery-induced sperm competition vary with water depth at which whitefish are caught
Source: Curr Zool. 2021 Jan 30;67(3):337–40. doi: 10.1093/cz/zoab007 (PMC8488998; doi:10.1093/cz/zoab007)
Supplement: zoab007_Supplementary_Data [file zoab007_supplementary_data.zip › Supplementary_Material_Perroud_et_al.pdf]

Supplementary Material

Male sexual signalling and expected effects of hatchery-induced sperm competition vary with water depth at which whitefish are caught

Giulia PERROUD, David NUSBAUMER, Christian de GUTTRY, Claus WEDEKIND*

Department of Ecology & Evolution, University of Lausanne, Lausanne, Switzerland

*Address correspondence to Claus Wedekind. E-mail: claus.wedekind@unil.ch

Handling editor: Murielle Ålund

Received on 4 September 2020; accepted on 18 January 2021

Key words: sexual signalling, sperm competition, whitefish

Materials and Methods

Adult whitefish (*Coregonus suidteri*, n = 104) were caught from Lake Hallwil (Switzerland; 47.2772° N, 8.2173° E) during their breeding season in January 2020 using gill nets set at 40, 25, 12, or 4 m depth along a line perpendicular to the bank (at decreasing distance to the shore, following the natural depth gradient). The nets were separated by distances of about 230, 50, and 40 m, respectively. At each depth, gill nets of 25, 27, 30, and 35 mm mesh size were used to ensure that adult whitefish of all size could be caught. Fish were immediately killed and kept in ice water until further processing. Standard body length (i.e. excluding the caudal fin) was determined, and scales were sampled above the lateral line from the caudal half of the body to later estimate fish age based on the number of annuli, a method offering same age estimates as methods based on otoliths or fin ray for fish younger than 5 years (Muir et al. 2008). Milt was collected by applying gentle bilateral abdominal pressure (see below). Fish were then scanned with a 3D optical scanner to determine mean breeding tubercles volume (see below).

For age estimation, scales were first cleaned using a cloth and 60% ethanol and then rinsed in water. Three scales by fish were mounted onto microscope slides and photographed under a 1.6x magnification. Fish were then aged based on the number of annuli, except for 1 female caught at 25 m depth for which we had no readable scales.

The milt of 22 males caught at 12 m and 4 m depths was stripped into large Petri dishes (145 × 20 mm, Greiner Bio-one, Frickenhausen, Germany; the discrepancy between this sample size and total males sample size (*n* = 71) was due to organizational problems in the field). Care was taken to strip the milt drop by drop and to separate theses drop on the Petri dish, so that all drops of milt that were non-contaminated with urine or faeces (i.e. usually all except the last drop) could be collected and individually pooled, 20 µL was stored at a dilution ratio of 1:9 in Storfish (IMV Technologies, l'Aigle, France), an isotonic inactivating medium, and kept on ice. The remaining milt was centrifugated to collect seminal plasma. Because the weight of the seminal plasma turned out to be not correlated to the sperm concentration (r = 0.18, d.f. = 18, p = 0.46), it was used to calculate the "milt potency", here defined as

milt potency = sperm concentration × weight of seminal plasma × average sperm velocity × maximum sperm longevity

The milt stored in Storfish were transported to the laboratory where sperm velocity and concentration were analysed within 48 h with CASA using the Qualisperm software (AKYmed AG, Cheseaux-sur-Lausanne, Switzerland) as in Nusbaumer et al. (2019), except that milt traits could mostly be summarised as a mean over 4 trials rather than over 2 trials. Briefly, 20 µL of each sample were activated in standardized water (OECD, 1992) at a 1:500 dilution ratio and measured at 20x magnification under phase contrast, at 6.5°C, 20s post-activation. Sperm longevity was measured as the time by which no more sperm motion could be observed. Our final sample size consisted of 20 males because the within sample measurement repeatability was too low in 2 samples (i.e. the standard error of sperm velocity and/or concentration exceeded 50% of the mean measurement; in the accepted samples, the standard errors of sperm velocity and concentration were on average 9 and 18 % of the means, respectively). The software also provided estimates for sperm motilities, i.e. percentage of activated sperm, but these estimates were not considered here because they showed low repeatability and included several unexplained outliers, i.e. motilities of < 30% while values >80% are usually expected (Sarosiek et al. 2016; Kowalski and Cejko 2019).

The average size of breeding tubercles per fish was determined with a 3D optical scanner (VR-5000, Keyence, Itasca, IL, USA). Each fish was first carefully dried before applying a thin layer of baby powder (Millette Baby powder, Migros, Zurich, Switzerland) on the skin to make the otherwise transparent breeding tubercles detectable by the optical scanner. The fish was then placed on a slightly angled plate so that its lateral line was approximately perpendicular to the laser beams in order to facilitate correction of the fish curvature in later analysis. Scans were then analysed using Keyence analysis software 3.1.0.56, with *curvature correction strength* set at 15, *reference plane* set as *continuous*, and measurements extracted using the *conplane method* to obtain the volume of individual breeding tubercles. All scans were carefully checked for anything that could potentially be wrongly identified as a breeding tubercle by the software.

Only 2 males and 4 females could be caught at 40 m depth. This depth category was therefore excluded from all statistical analyses. Also, because age classes varied from 1+ to 4+, but 89.5% of all fish where either 2+ or 3+, the fish were categorized as “young” (1+ or 2+, n=51) and “older” (3+ or 4+, n=44). Fisher’s exact test were then used for frequency analyses. An ANOVA was used to test for differences in body length between the catches. Because no differences in body length was found among the catches, a Gaussian generalised multiple regression analysis (GLM) was then used on the mean breeding tubercles volume as response variable to test the effects of depth at catch (specified as factor), body length, and the interaction of these two potential predictors (after graphically verifying that the model assumptions were not significantly violated). Analogous models were used to test whether depth at catch, body length, mean breeding tubercles volume, or any interaction between these factors would explain variance in mean sperm velocity and milt potency. The AIC of full models was compared to the AIC of models lacking a variable or an interaction and models with the lowest AIC were retained as final models. Wilcoxon rank sum tests were used on milt potency and components of milt potency to test whether they would vary between samples. The same method was used to test whether milt potency or components would vary between age categories. Analyses were done in RStudio 4.0.2 (R Development Core Team 2015) and JMP14.0.0 (SAS Institute Inc., Cary, NC).

References

Kowalski RK, Cejko BI, 2019. Sperm quality in fish: Determinants and affecting factors. *Theriogenology* **135**:94-108.

Muir AM, Sutton TM, Peeters PJ, Claramunt RM, Kinnunen RE, 2008. An evaluation of age estimation structures for lake whitefish in Lake Michigan: selecting an aging method based on precision and a decision analysis. *N Am J Fish Manag* **28**:1928-1940.

Nusbaumer D, Marques da Cunha L, Wedekind C, 2019. Sperm cryopreservation reduces offspring growth. *Proc R Soc B Biol Sci* **286**:20191644.

R Development Core Team. 2015. R: A language and environment for statistical computing. Vienna, Austria: R Foundation for Statistical Computing.

Sarosiek B, Dryl K, Judycka S, Dobosz S, Grudniewska J, et al., 2016. Cryopreservation method for whitefish (Coregonus lavaretus) semen possible for use in large-scale fertilization. *Aquaculture Research* **47**:4038-4042.

Table S1. Gaussian GLM on the effects of body length, depth at catch, and the interaction between the predictors on mean breeding tubercles volume. The full model had a lowest AIC, i.e. no factor or interaction was dropped during model selection. The tables give the estimated regression parameters, standard errors (SE), t-values, and p-values (N = 68).

Effect tested	Estimate	SE	*t*	*p*
Intercept	-0.48	0.1	- 4.3	< 0.001
Body length	0.28	0.3	1.0	< 0.001
Depth at catch = 12 m	0.03	0.0	5.3	< 0.001
Depth at catch = 4 m	0.52	0.1	3.6	< 0.001
Body length: depth at catch = 12 m	-0.03	0.0	-3.8	< 0.001
Body length: depth at catch = 4 m	-0.02	0.0	-1.2	0.32

Table S2. Gaussian GLM on the effects of depth at catch, mean breeding tubercles (BT) volume, body length, and potential interactions between the predictors on (a) sperm velocity and (b) milt potency. Model selection was based on AICs. The tables give the estimated regression parameters, standard errors (SE), t-values, and p-values (N = 20). The analogous model on sperm concentration could not be calculated because of significant differences of within-group variances (Bartlett $F_1 = 8.0$, $p < 0.01$). However, a graphical inspection of the data did not suggest significant effects of depth, body length, or breeding tubercles.

Effect tested	Estimate	SE	*t*	*p*
(a) Sperm velocity				
Intercept	-126.6	86.3	-1.5	0.16
Body length	11.4	3.9	2.9	0.01
(b) Milt potency				
Intercept	$1.8 \cdot 10^6$	$0.9 \cdot 10^6$	1.9	0.07
Depth at catch	$-1.5 \cdot 10^6$	$1.6 \cdot 10^6$	-1.0	0.34
BT mean volume	$-8.7 \cdot 10^6$	$11.1 \cdot 10^6$	-0.8	0.45
Depth at catch : BT mean volume	$45.2 \cdot 10^6$	$17.4 \cdot 10^6$	2.6	0.02

Table S3. Wilcoxon rank sum tests of difference in milt potency (in bold) and components of milt potency for (a) the two age groups, and (b) for fish caught at 12 m or 4 m depths.

Variable tested	W	*p*
(a) Fish age		
**Milt potency**	**31**	**0.61**
Sperm cells concentration	20	0.14
Mean sperm velocity	42	0.74
Weight of seminal plasma	40	0.87
Mean sperm longevity	35	0.83
(b) Depth		
**Milt potency**	**76**	**0.03**
Sperm cells concentration	59	0.43
Mean sperm velocity	48	1
Weight of seminal plasma	69	0.11
Mean sperm longevity	61	0.35

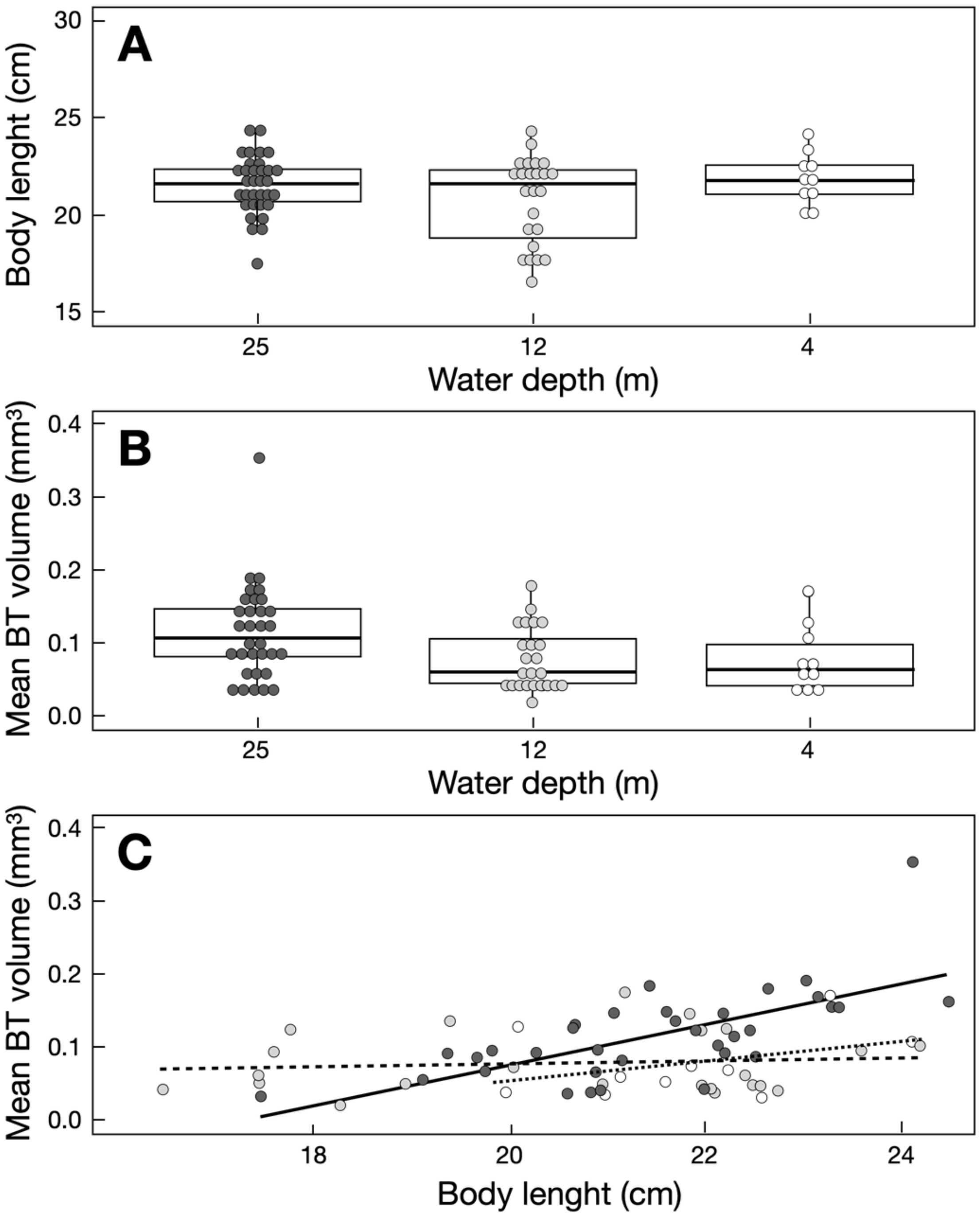

Figure S1. Phenotypes of males caught at different depths. (A) Body length (Tukey boxplots with quartiles and whiskers; ANOVA, F = 1.8, *df* = 2, *P* = 0.17), (B) mean breeding tubercles volume, and (C) relation between body length and breeding tubercles for fish caught at 25 m (dark grey symbols, solid regression line), at 12 m (grey symbols, dashed regression line) and at 4 m (open symbols, dotted line). BT: breeding tubercles. See Table S1 for statistics.
